# A Potential Diagnostic Role for a Combined Post-mortem DNA and RNA Sequencing for Brugada Syndrome

**DOI:** 10.1161/CIRCGEN.122.004251

**Published:** 2023-10-05

**Authors:** Carlos Bueno-Beti, David C. Johnson, Chris Miles, Joseph Westaby, Mary N. Sheppard, Elijah R. Behr, Angeliki Asimaki

**Affiliations:** Cardiovascular Clinical Academic Group, Molecular and Clinical Research Science Institute, St George’s University of London & St George’s University Hospital NHS Foundation Trust, London, United Kingdom

**Keywords:** FFPE samples, DNA sequencing, RNA sequencing, Brugada Syndrome, postmortem, diagnosis

Post-mortem genetic testing (molecular autopsy) is an important tool to identify genetic risk in family members following an unexplained sudden death (sudden arrhythmic death syndrome - SADS). However, exome sequencing is currently informative in only 13-30% of cases.^1^ RNA sequencing (RNAseq) has been shown to aid genetic diagnosis where DNA sequencing (DNAseq) is uninformative. Formalin-fixed paraffin-embedded (FFPE) heart tissue is retained routinely for histopathological examination after sudden cardiac death (SCD). Unfortunately, FFPE processing can lead to fragmentation, DNA crosslinks and deamination leading to false positives variant calling in the subsequent sequencing. Brugada Syndrome (BrS), a heritable arrythmia syndrome, is the most common underlying cause of death in SADS.^1^ One gene, *SCN5A*, has definitive evidence for disease causation, but only underlies approximately 20% of clinical cases,^2^ hampering the potential role for molecular autopsy as a diagnostic tool. We aimed to demonstrate that the combination of the DNAseq and RNAseq in post-mortem tissue can successfully identify putative causative variants and establish whether there may be a distinctive functional expression profile in the RVOT of BrS decedents.

Six BrS cases with an antemortem diagnosis retrieved from the medical record employing expert consensus and Shanghai scoring criteria^3^ and 5 age and sex-matched controls with a non-cardiac death were selected for this study. FFPE heart tissue from the RVOT, and where available, suitable samples of splenic tissue, were obtained from the Cardiac Risk in the Young Centre for Cardiac Pathology at St. George’s, University of London. DNA and RNA were extracted from the FFPE heart samples following manufacturer’s instructions and their integrity determined with Agilent Tape Station. DNA and RNA sequencing were undertaken on an Illumina HiSeq instrument. Sequence adapters were removed from 2x150 paired end RNA sequencing with Trimmomatic v0.39. Alignment was undertaken with STAR-2.7.3a onGRCh38. QC metrics were assessed by FastQC, QualiMap, RNASeqMetrics and PICARD. FeatureCounts generated counts for each gene. To call variants, SplitNCigarReads, BaseRecalibrator, ApplyBQSR and HaplotypeCaller were applied to aligned DNA-seq and RNA-seq in accordance with germline short variant discovery GATK (v4) guidance. Overall, 198 unique genes were investigated including: Sudden Cardiac Death (n=87); Brugada Syndrome (n=23); and Trusight Cardio (n=172). Differential expression between BrS cases and Control subjects was assessed using DeSeq2.27 (false discovery rate cut-off at 0.01). Gene set enrichment analysis (GSEA) was performed using all genes ranked by their differential mRNA expression. Ethical approval was granted by the London Stanmore National Health Service Research Ethics Committee (reference: 10/H0724/38).

Variant calling on DNAseq data revealed two *SCN5A* variants, p.S1315X in case B4 and p.T17Ile in case B6 classified as pathogenic and likely pathogenic respectively by ACMG criteria. Additionally, we observed 19 VUSs in 14 different genes (gnomAD allele frequency (AF) ≤ 10-4, popmax filtering AF < 1.85 x 10-4 and reads ≥ 20; Figure 1).

Variant calling on RNAseq data (gnomAD AF ≤ 10-5, CADD > 20 and RNA reads ≥ 20) confirmed the presence of *SCN5A:* p.S1315X and *SCN5A:* p.T17I in the transcriptome. Only 5 out of the 19 VUSs in genes *FKRP*, *JUP*, *TRIM63*, *RYR2* and *TTN* identified in DNAseq data were detected in the RNAseq data set. The subjects were heterozygous for all the variants identified in this study. Three genes at loci previously demonstrated as genome-wide significant associated with BrS, *SCN5A, IRX3* and *IRX5*, showed significant differential expression between BrS and controls, regardless of the presence of a *SCN5A* variant (Figure).

Gene set expression analysis revealed 50 novel genetic associations with BrS. Interestingly,13 of these 50 new associations are present in two liver specific gene sets (NES = 4.06 and 3.30 with FDR = 0; Figure).

The data that support the findings of this study are available from the corresponding author upon reasonable request.

By merging variant calling data from DNAseq with RNA-seq from the same tissue source, an improved diagnostic accuracy of variant calling can be achieved in molecular autopsy. Gene expression analysis of RNAseq data from FFPE heart samples demonstrated reduced SCN5A expression levels in all BrS patients, regardless of *SCN5A* genotype. Furthermore, we associated 50 novel genes, including liver specific gene sets, with BrS that could be used as an expression profile of the disease with the potential for improving the diagnostic accuracy and yield in SADS decedents. Interestingly, the most strongly associated liver gene, alcohol dehydrogenase1B (*ADH1B*), has been previously associated with arrhythmic events after alcohol drinking in a BrS cohort.^4^

The transcriptional and post-transcriptional regulation of *SCN5A* in myocardial tissue may determine the penetrance and expressivity of associated diseases such as BrS. IRX3 and IRX5, two well-known regulators of the expression of different ion-channels in the adult heart showed reduced expression in BrS decedents. *SCN5A, IRX3* and *IRX5* have proximal SNP variants associated with BrS with genome-wide significance, suggesting that the regulation of these genes is important in BrS risk.^5^

This study therefore supports the potential utility of combining RNA-seq with DNA-seq of FFPE tissue of SCD decedents in a novel approach to molecular autopsy that requires further prospective investigation. It also unveils genomic pathways adding to the risk of BrS.

## Figures and Tables

**Figure 1:**
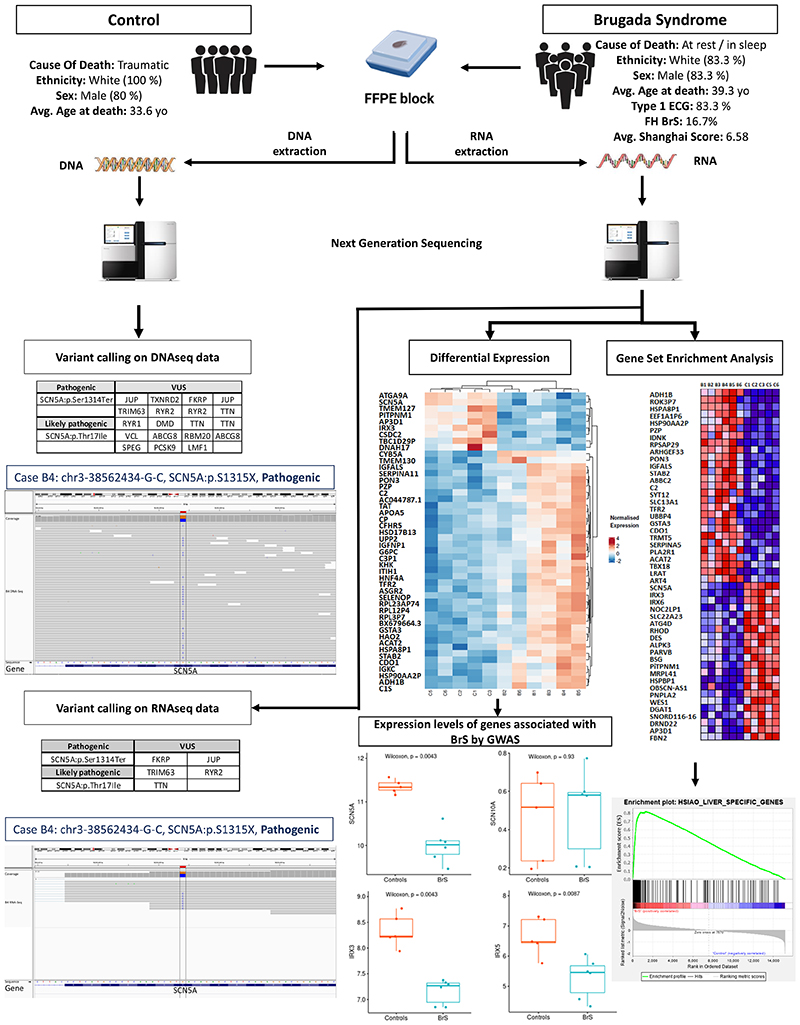
Combined DNA and RNA sequencing analysis approach as a potential diagnostic tool in Brugada Syndrome. FFPE heart tissue from the RVOT from 6 Brugada Syndrome patients and 5 age and sex-matched controls with a non-cardiac death, were selected for this study. DNA and RNA from all samples was extracted and sequenced on an Illumina HiSeq instrument. Alignment was undertaken with STAR-2.7.3a on GRCh38. QC metrics were assessed by FastQC, QualiMap, RNASeqMetrics and PICARD. FeatureCounts generated counts for each gene. To call variants, SplitNCigarReads, BaseRecalibrator, ApplyBQSR and HaplotypeCaller were applied to aligned DNA-seq and RNA-seq in accordance with germline short variant discovery GATK (v4) guidance. A total of 198 genes were investigated. Differential expression between BrS cases and Control subjects was assessed using DeSeq2.27 (false discovery rate cut-off at 0.01). Gene set enrichment analysis (GSEA) was performed using all genes ranked by their differential mRNA expression.

## Nonstandard Abbreviations and Acronyms

AF: Allele frequency
BrS: Brugada syndrome
DNAseq: DNA sequencing
FFPE: Formalin-fixed paraffin-embedded
gnomAD: Genome Aggregation Database
GSEA: Gene set enrichment analysis
RNAseq: RNA sequencing
RVOT: Right ventricular outflow tract
SADS: Sudden arrhythmic death syndrome
SCD: Sudden cardiac death
*SCN5A*: Sodium Voltage-Gated Channel Alpha Subunit 5
VUS: variants of uncertain significance
